# Renal Artery Aneurysm Rupture as a Dangerous Mimic of Ovarian Cyst Rupture: A Case Report

**DOI:** 10.5811/cpcem.1585

**Published:** 2024-05-14

**Authors:** Lauren Kaplan, Kaushal H. Shah, Christie Lech, Mary-Kate Gorlick

**Affiliations:** * New York-Presbyterian Hospital, New York, New York; † New York-Presbyterian Hospital; Weill Cornell Medical Center, Department of Emergency Medicine, New York, New York

**Keywords:** *case report*, *aneurysm*, *renal*, *rupture*

## Abstract

**Introduction:**

Renal artery aneurysm rupture is a rare but morbid diagnosis, often requiring emergency surgery and nephrectomy. Clinical presentation can mimic more common pathology in non-pregnant women such as ruptured ovarian cyst.

**Case Report:**

We present a case of a woman with a prior history of ovarian cyst presenting with a ruptured renal artery aneurysm. Prompt computed tomography (CT) imaging revealed a left renal artery aneurysm rupture with hemoperitoneum and renal infarct. She underwent emergency laparotomy and nephrectomy and was ultimately discharged in good condition.

**Conclusion:**

While ovarian cyst rupture is the most common cause of spontaneous hemoperitoneum in non-pregnant women of childbearing age, renal artery aneurysm rupture should be considered and prompt CT imaging obtained, particularly in cases of hemodynamic instability, to ensure prompt treatment.

Population Health Research Capsule What do we already know about this clinical entity?

*Renal artery aneurysm (RAA) rupture is a rare but morbid diagnosis that can lead to emergency surgery and nephrectomy.*

What makes this presentation of disease reportable?

*We report a case of spontaneous ruptured RAA presenting as a dangerous mimic of ovarian cyst rupture, a more common pathology in non-pregnant women.*

What is the major learning point?

*
Emergency physicians should maintain an index of suspicion for emergent vascular pathology in non-pregnant women of childbearing age with spontaneous hemoperitoneum.*

How might this improve emergency medicine practice?

*Maintaining an index of suspicion for rare disease processes such as RAA rupture will ensure prompt recognition and treatment in the ED.*

## INTRODUCTION

Renal artery aneurysm (RAA) is a rare diagnosis, estimated to occur in 0.09% of the general population.^5^ Rupture is a rare but morbid complication, often requiring emergent surgery and nephrectomy. In contrast, ovarian cyst rupture is the most common cause of spontaneous hemoperitoneum in non-pregnant women of reproductive age and is usually managed conservatively in the absence of hemodynamic compromise or associated torsion.^1^ ^,^ ^2^ We report a case of spontaneous ruptured RAA as a dangerous mimic of ovarian cyst rupture.

## CASE REPORT

A 52-year-old woman with a past medical history of ovarian cysts was brought in by emergency medical services (EMS) to our emergency department (ED) for acute onset of atraumatic left lower quadrant pain. Her symptoms started abruptly while at work, which she stated felt like symptoms one year prior when she was found to have ovarian cysts and likely an ovarian cyst rupture. She endorsed lightheadedness, but she denied any shortness of breath, chest pain, cough, fevers, chills, or changes in urination.

Per EMS report, she acutely became pale and somnolent, associated with bradycardia to the 40s. In the ED she was hypotensive to 84/48 millimeters of mercury, with heart rate 84 beats per minute, oxygen saturation 100% on room air, and she was afebrile. Physical examination revealed pallor, somnolence, cool extremities, and a peritoneal abdomen. Focused assessment with sonography in trauma examination was positive for free fluid in the left upper quadrant. Pregnancy test was negative. Initial labs were notable for lactate 4.59 millimoles per liter (mmol/L) (reference range 0.50–1.60 mmol/L). Initial hemoglobin was 10 grams per deciliter (g/dL) (12.6–17.0 g/dL); hematocrit 30.5% (37.2–47.9%); platelets 317,000 per milliliter (mL) (156,000–325,000/mL); and white blood cell count 15,000/mL (3,120–8,440/mL). Coagulation factors were normal. Chemistry panel was notable for sodium 130 mmol/L (137–145 mmol/L); potassium 3.3 mmol/L (3.5–5.1 mmol/L); bicarbonate 21 mmol/L (19–27 mmol/L); blood urea nitrogen 18 milligrams (mg)/dL (7–26 mg/dL); and creatinine 1.0 mg/dL (0.70–1.30 mg/dL). Emergency physicians activated a massive transfusion protocol and paged the obstetrics and gynecology service due to concern for hemorrhagic ruptured ovarian cyst vs ovarian torsion.

Emergent computed tomography of the abdomen and pelvis revealed a large left retroperitoneal and peritoneal hematoma secondary to left RAA rupture, as well as concern for developing splenic infarcts in the left lower renal pole (Image). The patient was taken emergently to the operating room (OR) for exploratory laparotomy within two hours of ED arrival. She underwent suprarenal cross-clamping with repair of the left renal artery and ligation of renal vessels. She returned to the OR two days later for left nephrectomy and abdominal closure. She was extubated and transferred to the floor. She was discharged home two days later in good condition.

**Image.**
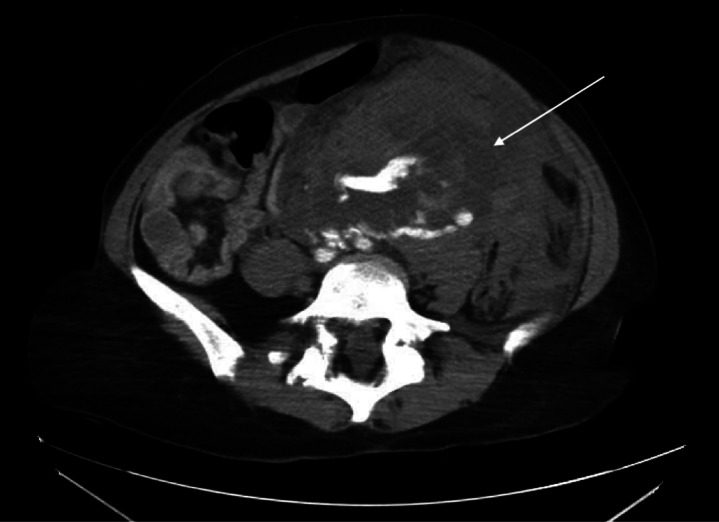
Cross-sectional image of abdominal computed tomography showing hematoma with extravasation of contrast (arrow) as a result of ruptured left renal artery aneurysm.

## DISCUSSION

Although the incidence of RAA is rare, ranging from 0.01–0.09% of the population, this case report illustrates the importance of timely diagnosis.^3^ ^,^ ^4^ Contemporary rupture rates are estimated at approximately 3%.^5^ They are most commonly found in women >60 years with risk factors including hypertension, fibrodysplasia, and connective tissue disorders causing arterial medial wall degeneration. Patients notably lack traditional cardiovascular risk factors such as cigarette use and diabetes.^6^ ^,^ ^7^ Aneurysms are usually asymptomatic and found incidentally on screening imaging, although patients can present with symptoms such as hypertension, flank pain, hematuria, and abdominal pain.^5^

As the presentation of a ruptured RAA can be identical to the more common ruptured ovarian cyst, consideration of rare serious surgical pathology should be maintained for patients with acute abdominal pain and free fluid on exam. Bradycardia in the setting of hemoperitoneum is a well described phenomenon particularly in ruptured ectopic pregnancy and can indicate hemorrhagic shock, both of which were considerations in the case reported here.^8^ ^,^ ^9^ Other emergent complications of RAA include thrombosis, embolism, and obstructive uropathy.

For non-ruptured RAA, surgical or endovascular intervention is recommended for aneurysms with a diameter exceeding two centimeters, patients with uncontrolled symptoms (ie. pain or refractory hypertension), or in women of childbearing age (as there are higher rates of rupture and mortality in pregnancy and puerperium than in the general population).^3^ ^,^ ^6^ These guidelines remain somewhat controversial given the increased incidence of aneurysms uncovered by widespread use of imaging combined with a knowledge gap of the natural progression of disease.^2^ ^,^ ^4^ Endovascular intervention through stenting or angioembolization is a safe and effective alternative to open repair, with studies suggesting a trend toward shorter hospital stays and fewer complications.^10^ In cases of hemodynamic compromise, exploratory laparotomy and nephrectomy are often indicated.^2^ ^,^ ^3^ ^,^ ^4^

## CONCLUSION

This is a case of spontaneous rupture of a left renal artery aneurysm as a dangerous mimic of ovarian cyst rupture. While ovarian cyst rupture remains the most common cause of spontaneous hemoperitoneum in non-pregnant women of childbearing age, RAA should be considered with confirmation via computed tomography, particularly in cases of hemodynamic instability to ensure prompt treatment of a disease with potentially high morbidity and mortality.
